# Sialyl-Tn glycan epitope as a target for pancreatic cancer therapies

**DOI:** 10.3389/fonc.2024.1466255

**Published:** 2024-09-13

**Authors:** Rafaela Abrantes, Joanne Lopes, Daniel Lopes, Joana Gomes, Sónia A. Melo, Celso A. Reis

**Affiliations:** ^1^ Instituto de Investigação e Inovação em Saúde (i3S), Universidade do Porto, Porto, Portugal; ^2^ Institute of Molecular Pathology and Immunology of the University of Porto (IPATIMUP), Porto, Portugal; ^3^ Instituto de Ciências Biomédicas Abel Salazar (ICBAS), Universidade do Porto, Porto, Portugal; ^4^ Department of Pathology, Unidade Local de Saúde (ULS) de São João, Porto, Portugal; ^5^ Faculty of Medicine of the University of Porto (FMUP), Porto, Portugal; ^6^ Porto Comprehensive Cancer Center (P.CCC), Porto, Portugal

**Keywords:** Sialyl-Tn, glycosylation, pancreatic cancer, targeted therapy, truncated *O*-glycans

## Abstract

Pancreatic cancer (PC) is the sixth leading cause of cancer-related deaths worldwide, primarily due to late-stage diagnosis and limited treatment options. While novel biomarkers and immunotherapies are promising, further research into specific molecular targets is needed. Glycans, which are carbohydrate structures mainly found on cell surfaces, play crucial roles in health and disease. The Thomsen-Friedenreich-related carbohydrate antigen Sialyl-Tn (STn), a truncated *O*-glycan structure, is selectively expressed in epithelial tumors, including PC. In this study, we performed a comprehensive analysis of STn expression patterns in normal, premalignant, and malignant pancreatic lesions. Additionally, we analyzed the association between STn expression and various clinicopathological features. STn expression was statistically associated with pathological diagnosis; it was absent in normal pancreatic tissue but prevalent in pancreatic carcinoma lesions, including pancreatic ductal adenocarcinoma (PDAC), pancreatic acinar cell carcinoma, and pancreatic adenosquamous carcinoma. Moreover, we found a significant association between STn expression and tumor stage, with higher STn levels observed in stage II tumors compared to stage I. However, STn expression did not correlate with patient survival or outcomes. Furthermore, STn expression was assessed in PDAC patient-derived xenograft (PDX) models, revealing consistent STn levels throughout engraftment and tumor growth cycles. This finding supports the PDX model as a valuable tool for testing new anti-STn therapeutic strategies for PC in clinical setting.

## 1 Introduction

Pancreatic cancer (PC) ranks as the sixth leading cause of cancer-related mortality worldwide, with 511,000 new cases and 467,000 deaths reported in 2022 (1). Over the last decades, the significance of PC as a public health concern has increased, while mortality rates for other common cancers, including gastric, colorectal, prostate, and breast cancers, have concurrently declined (1). Despite significant advances in cancer therapy, PC remains a challenging disease characterized by late-stage diagnosis and limited therapeutic options, resulting in a 5-year survival rate of only 12% (2). Currently, surgical resection followed by adjuvant chemotherapy is the sole potentially curative therapy available (3, 4). However, because PC is typically asymptomatic and there is a lack of biomarkers with sufficient specificity and sensitivity, most patients present with locally advanced or metastatic disease, rendering them ineligible for surgical intervention (3, 4). Emerging biomarkers and novel immunotherapeutic approaches, such as antibody-drug conjugates (ADCs) and Chimeric Antigen Receptor (CAR) T cells, hold promise for the future of PC treatment.

Glycans, carbohydrate structures attached to a variety of carrier molecules predominantly found on the surface of mammalian cells, play crucial cellular roles and have profound implications in both health and disease (5). Alterations in the glycan profiles of cancer cells have been extensively documented, often correlating with the aggressiveness and progression of cancer (6, 7). In the context of PC, glycan structures such as sialylated and fucosylated Lewis blood group antigens, including Sialyl Lewis a (SLe^a^) and Sialyl Lewis x (SLe^x^), and short *O*-glycans like the Tn and Sialyl-Tn (STn) antigens, are known to be upregulated and have been associated with increased invasion and metastasis (8–13). SLe^a^, detectable by the CA-19.9 monoclonal antibody (mAb), is the only FDA-approved blood-based tumor biomarker for monitoring PC (14). However, the presence of SLe^a^ in benign conditions limits its diagnostic application, primarily confining it to a prognostic tool for managing late-stage disease (14). In contrast, STn (NeuAcα2–6-GalNAcα1-O-Ser/Thr), a truncated sialylated *O*-glycan structure, exhibits selective expression in epithelial tumors, including PC, while being notably absent in normal tissues (10–13, 15–21). Additionally, STn has been shown to play an important role in the malignant phenotype of cancer cells (12, 13, 22, 23). This distinctive profile positions STn as a promising candidate for both cancer biomarker and therapeutic target (24–42). Despite extensive research, accurately mapping and targeting of STn has been hindered by variations in the epitope binding features of available mAbs (39, 43, 44). Most studies on STn in PC have focused on its detection using HB-STn1 (clone 3F1) and TKH2 mAbs, with limited reports employing the B72.3 mAb (10, 11, 17, 45–48). The B72.3 mAb, generated by mice immunization with human breast carcinoma cells, was the first anti-STn mAb described and has been widely used in both tissue-based and serological assays, as well as in preclinical studies (49–53).In this work, we address this research gap by performing a comprehensive study on the expression patterns of STn in normal, premalignant, and malignant lesions of the pancreas using the B72.3 mAb. We further analyzed the association between STn expression and clinicopathological features of PC patients to understand its prognostic and therapeutic potential. Additionally, STn expression was assessed in pancreatic ductal adenocarcinoma (PDAC) patient-derived xenografts (PDXs), a potential tool for testing new anti-STn therapeutic approaches for PC.

## 2 Materials and methods

### 2.1 Clinical samples

Pancreatic tissue microarrays (TMAs) were obtained from US Biomax, Rockville, MD, USA. TMAs used in this study, BIC14011b and PA2082a, collectively comprised 14 normal pancreatic tissues, 15 premalignant lesions of the pancreas, and 78 pancreatic tumor cores, each case spotted in duplicate. Exclusion criteria included no available tumor tissue sample adequate for STn expression evaluation, or the absence of clinicopathological data. Patient’s specifications, including age, gender, pathological diagnosis, tumor stage, and the presence of lymph node metastasis were obtained from the US Biomax Inc. database (https://www.biomax.us/).

A retrospective cohort of PDAC (n=39) was obtained from the biobank of the *Unidade Local de Saúde* (ULS) *de São João*, Porto, Portugal. The collection and handling of these samples were performed in accordance with national regulatory laws governing the handling of biological specimens. This study was conducted in strict compliance with ethical guidelines and Helsinki declaration and approved by the Centro Hospitalar Universitário de São João (CHUSJ) and FMUP ethics committee (CES 253/22). Clinicopathological information was obtained from patients’ clinical records.

### 2.2 Immunohistochemistry

Formalin-fixed paraffin-embedded (FFPE) tissue sections from PDAC cohort and tissue microarrays were used. The expression of the short *O*-glycan STn detected using the B72.3 mAb was analyzed by immunohistochemistry (IHC). For the tissue microarrays, an initial baking step for 1h at 60°C was performed to prevent tissue detachment. Deparaffinization and rehydration of tissue sections was done, followed by neutralization of endogenous peroxidase activity with 3% hydrogen peroxide. For blocking of non-specific background staining, the UltraVisionTM Protein Block (Thermo Fisher Scientific, Waltham, MA, USA) was used for 5 min at room temperature (RT). Tissue sections were incubated overnight (ON) at 4°C with the anti-STn mAb B72.3 diluted in 5% bovine serum albumin (BSA)/phosphate-buffered saline (PBS) (dilution 1:2), followed by incubation with goat anti-mouse biotin-labeled secondary antibody (Dako, Berlin, Germany) diluted in 5% BSA/PBS (dilution 1:200) for 30 min at RT. Tissue sections underwent chromogenic staining with 3,3′-diaminobenzidine (DAB, Sigma-Aldrich, St. Louis, MO, USA) supplemented with 0.01% hydrogen peroxide, and nuclear staining was performed with Mayers’ hematoxylin. Controls were included in every set of slides, with normal gastric mucosa tissues serving as negative controls and intestinal metaplasia tissues as positive controls. The IHC staining of each tissue core was blindly evaluated and scored by a board-certified pathologist with vast experience in gastrointestinal pathology, using a semi-quantitative methodology. Evaluation of STn expression involved both the percentage of stained cells and the localization of the staining within the tissue. For statistical analysis, STn positivity was defined as staining present in more than 5% of the cells. The staining patterns were classified as membranous, cytoplasmic, or a combination of both. Representative images illustrating STn staining pattern were selected by the specialist pathologist.

### 2.3 Patient-derived xenografts

Patient-derived xenografts from PDAC were obtained as previously described (54). Briefly, PDAC patient-derived tumor cells were orthotopically implanted in the pancreas of immunodeficient mice (Rag2^−/−^Il2rg^−/−^) (54). Tumor growth was monitored by ultrasound (MicroUltrasound Vevo 2100, RRID: SCR_015816). When tumor size reached 1500 mm^3^ or mice presented severe symptoms, mice were euthanized and tumors fixed in neutral buffered formalin, embedded in paraffin, and processed for IHC staining using the B72.3 mAb, as described above. This study was approved by the CHUSJ and FMUP ethics committee (2015-12-11).

### 2.4 Statistical analysis

Statistical analysis was performed using Statistical Package for the Social Sciences (SPSS, IBM Inc., New York, NY, USA) version 25. Categorical variables were described as absolute and relative frequencies, and continuous variables were designated using median values. Categorical variables were compared using the Chi-square test or Fisher exact test, as appropriate. Multivariate analysis was performed using Cox Regression. Differences were considered statistically significant if *p*<0.05.

## 3 Results

### 3.1 Patient’s clinicopathological characteristics

The retrospective cohort consisted of 146 individuals, including 84 males (57.5%) and 62 females (42.5%), with a median age of 57 years (range 21-81 years). The cohort comprised 14 individuals (9.6%) with normal pancreatic tissue, 15 (10.3%) with premalignant pancreatic lesions, and 117 (80.1%) diagnosed with PC. Among those with precursor lesions, 7 patients (4.8%) had chronic pancreatitis, 3 patients (2.1%) exhibited pancreatic intraepithelial neoplasia (PanIN) 1, 3 patients (2.1%) had PanIN 2, and 2 patients (1.4%) presented PanIN 3 lesions. This study encompassed patients with various subtypes of PC, with the majority having PDAC (112 patients, 76.7%). Additionally, 2 patients (1.4%) were diagnosed with pancreatic acinar cell carcinoma, and 1 patient each (0.7%) with pancreatic squamous cell carcinoma, adenosquamous carcinoma, and neuroendocrine carcinoma. Detailed clinicopathological parameters of the individuals included in this study are presented in **Table 1**.

**Table 1:** Association between clinicopathological parameters and Sialyl-Tn positivity.

Clinicopathological parameters		Total	STn negative	STn positive	*p* value^1^
		n (%)	n (%)	n (%)	
		146 (100)	72 (49.3)	74 (50.7)	
Gender	Male	84 (57.5)	42 (50.0)	42 (50.0)	0.847
	Female	62 (42.5)	30 (48.4)	32 (51.6)	
Age of diagnosis	≤57 years	73 (50.0)	40 (54.8)	33 (45.2)	0.185
	>57 years	73 (50.0)	32 (43.8)	41 (56.2)	
Pathological diagnosis	Normal pancreas	14 (9.6)	14 (100)	0 (0)	<0.001^*^
	Premalignant lesions	15 (10.3)	13 (86.7)	2 (13.3)	
	Chronic pancreatitis	7 (4.8)	7 (100)	0 (0)	
	PanIN 1	3 (2.1)	2 (66.7)	1 (33.3)	
	PanIN 2	3 (2.1)	3 (100)	0 (0)	
	PanIN 3	2 (1.4)	1 (50.0)	1 (50.0)	
	Pancreatic cancer	117 (80.1)	45 (38.5)	72 (61.5)	
	Acinar cell carcinoma	2 (1.4)	1 (50.0)	1 (50.0)	
	Squamous cell carcinoma	1 (0.7)	1 (100)	0 (0)	
	Adenosquamous cell carcinoma	1 (0.7)	0 (0)	1 (100)	
	Neuroendocrine carcinoma	1 (0.7)	1 (100)	0 (0)	
	PDAC	112 (76.7)	42 (37.5)	70 (62.5)	
Tumor stage (n=117^**^)	I	34 (29.1)	18 (52.9)	16 (47.1)	0.035^*^
	II	80 (68.4)	25 (31.2)	55 (68.8)	
	III	2 (1.7)	2 (100)	0 (0)	
	IV	1 (0.9)	0 (0)	1 (100)	
Lymph node metastasis (n=117^**^)	Absent	80 (68.4)	32 (40.0)	48 (60.0)	0.615
	Present	37 (31.6)	13 (35.1)	24 (64.9)	

PanIN, pancreatic intraepithelial neoplasia; PDAC, pancreatic ductal adenocarcinoma; STn, Sialyl-Tn. ^*^Statistical significant results (p<0.05). ^**^Number of patients with pancreatic cancer. ^1^Pearson chi-squared test, Fisher’s Exact Test, and Independent Samples T Test (age of diagnosis).

### 3.2 Sialyl-Tn expression in pancreatic tissues

**Figure 1:**
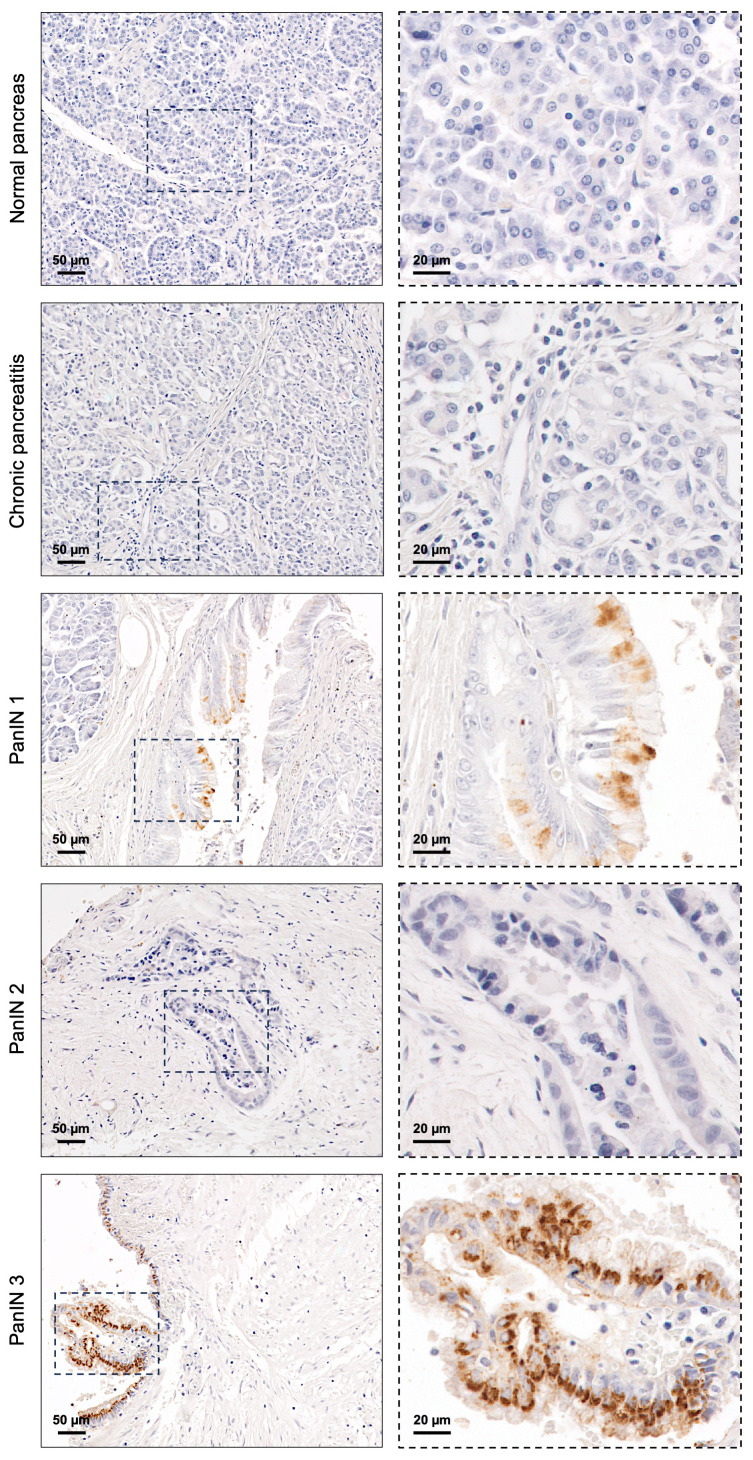
Sialyl-Tn expression in normal pancreatic tissue and premalignant lesions of the pancreas. Normal pancreatic tissue, chronic pancreatitis lesion, and pancreatic intraepithelial neoplasia (PanIN) 2 lesion without Sialyl-Tn (STn). PanIN 1 and PanIN 3 lesions with cytoplasmic STn expression.

The expression of the STn glycan antigen was evaluated in the entire cohort, comprising normal, premalignant, and PC tissues, using the B72.3 mAb. STn was not detected in any of the 14 normal pancreatic tissues tested (**Figure 1**; **Table 1**). Among the 15 premalignant lesions, STn expression was observed in PanIN lesions (13.3%) (**Table 1**), with exclusive cytoplasmic staining (**Figure 1**). No STn expression was identified in chronic pancreatitis (**Figure 1**).

**Figure 2:**
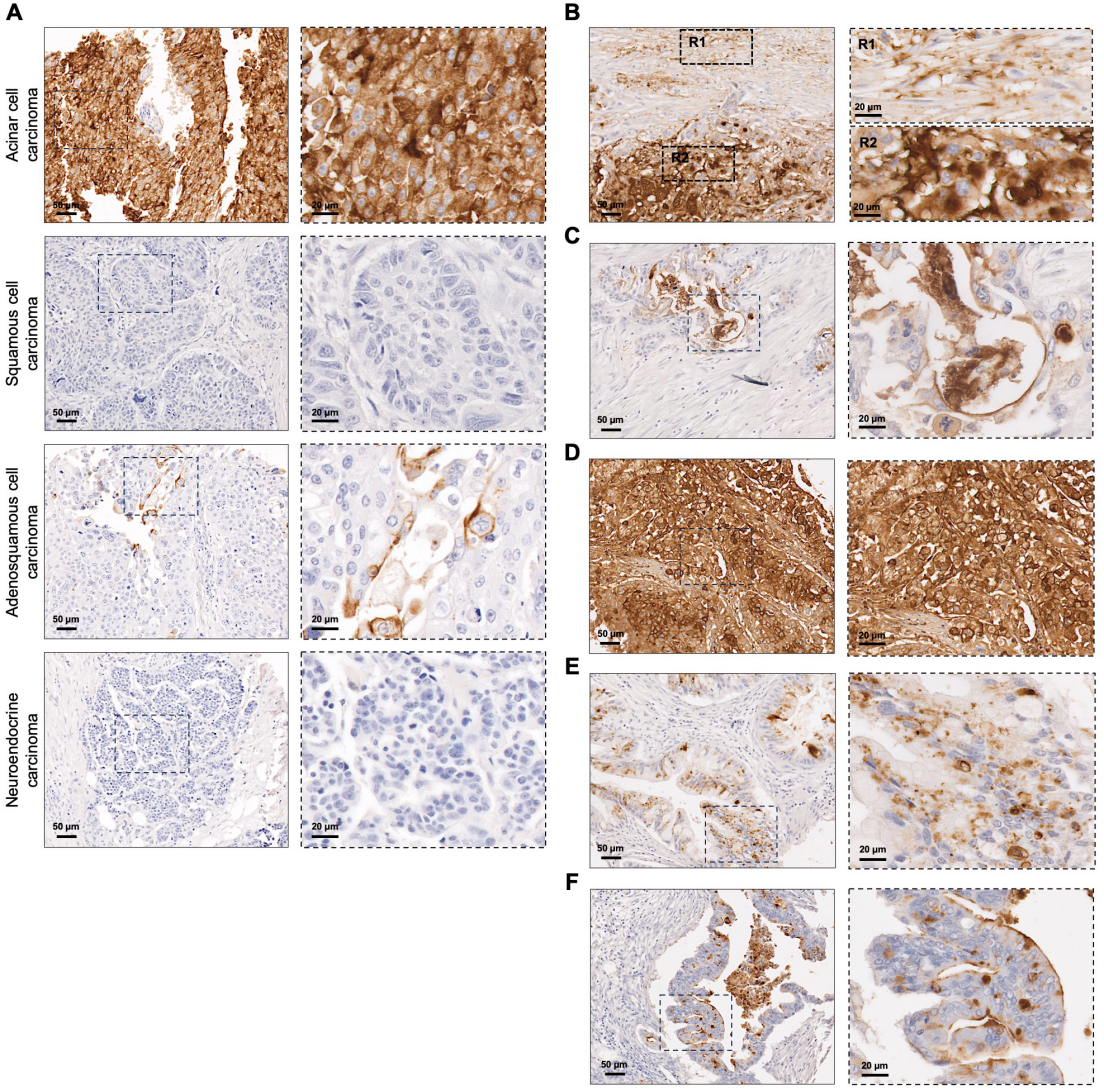
Sialyl-Tn expression in pancreatic cancer. **(A)** Screening of Sialyl-Tn (STn) within rare subtypes of pancreatic cancer (PC) revealing its presence in acinar and adenosquamous cell carcinomas, and absence in squamous cell and neuroendocrine carcinoma lesions. **(B–F)**. STn mapping in pancreatic ductal adenocarcinoma (PDAC). **(B)** PDAC lesion with heterogeneous STn expression. Two regions (R1-2) were selected: R1- tumor with moderate STn expression; R2- tumor with high STn expression. **(C)** PDAC with moderate STn expression levels. **(D)** PDAC lesion with high and homogeneous STn expression. **(E)** PDAC with cytoplasmic STn expression. **(F)** PDAC lesion with both cytoplasmic and membranous STn staining.

In the 117 PC tissues analyzed, STn was detected in 72 lesions (61.5%) (**Table 1**). This group included rare PC subtypes, such as pancreatic acinar cell carcinoma, squamous cell carcinoma, adenosquamous cell carcinoma, and neuroendocrine carcinoma. Notably, one case of pancreatic acinar cell carcinoma exhibited 100% STn expression, with both membranous and cytoplasmic staining (**Figure 2A**). The pancreatic adenosquamous carcinoma case showed low STn expression, primarily localized to the cell membrane, while the pancreatic squamous cell carcinoma and neuroendocrine carcinoma cases completely lacked STn expression (**Figure 2A**). Among the total cases of PDAC (n=112), over half of the cases (62.5%) expressed STn (**Table 1**). Importantly, STn positivity was consistent in PDAC among the different cohorts studied (data not shown). Additionally, heterogeneity in STn expression was observed within PDAC, both within individual tumor lesions (**Figure 2B**) and among different tumors. Some PDAC cases exhibited moderate STn levels (**Figure 2C**), while others showed a very strong and uniform staining for STn (**Figure 2D**). In terms of STn staining patterns within PDAC lesions, some cases displayed exclusively cytoplasmic staining (**Figure 2E**), whereas others had STn presence both in the cytoplasm and on the cell membrane (**Figure 2F**).

The expression of STn was associated with clinicopathological parameters (**Table 1**). As expected, a significant association was found between STn expression and pathological diagnosis (*p*<0.001). STn was absent in normal pancreatic tissues, present in few premalignant pancreatic lesions (13.3%), and highly prevalent in pancreatic carcinoma (**Table 1**). Specifically, 61.5% of pancreatic carcinoma lesions tested positive for STn. There was no association between STn expression and gender or age. Among individuals with PC lesions (n=117), an association was observed between tumor stage and STn status (*p*=0.035), with higher STn expression noted in stage II tumors compared to stage I. However, no correlation was found between STn levels and the presence of lymph node metastasis (*p*=0.615).

### 3.3 Patient outcomes

Patient outcomes were assessed in a subset of 39 individuals diagnosed with PDAC. The median follow-up time was 16.9 months (range 0.4-151.5 months). During this period, 28 patients (71.8%) died. The median overall survival (OS) was 16.4 months (range 0.4-147.1 months). As expected, patients without lymph node metastases had significantly better OS compared to those with lymph node involvement (*p*=0.037). No significant correlation was observed between OS and gender (*p*=0.605), age (*p*=0.676), or tumor stage (*p*=0.339). Additionally, in this exploratory cohort, no association was found between STn expression and patient outcome.

### 3.4 Sialyl-Tn expression validation in patient-derived xenografts

**Figure 3:**
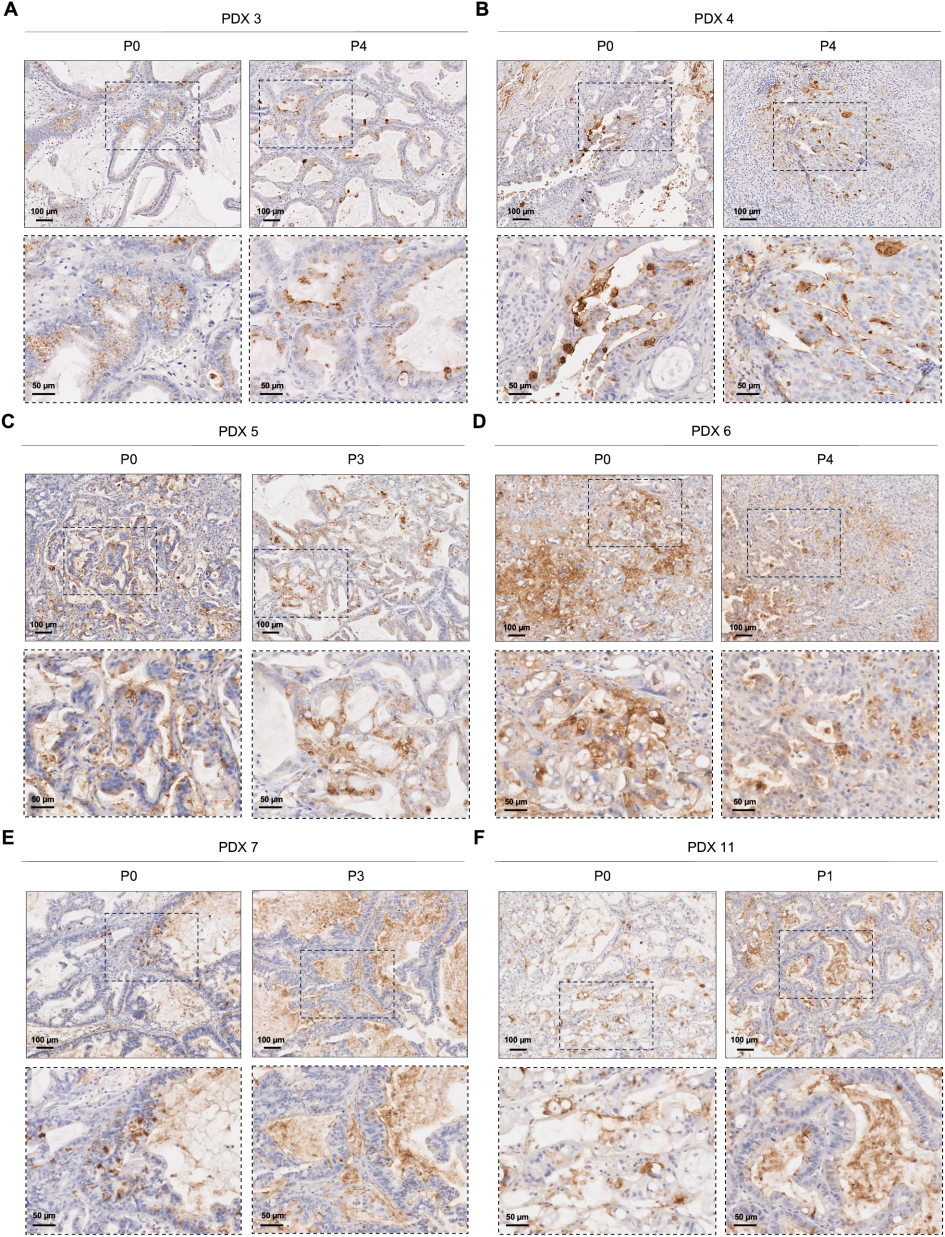
Sialyl-Tn expression in pancreatic ductal adenocarcinoma patient-derived xenografts. **(A)** Patient-derived xenografts (PDXs) with heterogeneous cytoplasmic Sialyl-Tn (STn) expression in both tested passages (P). **(B)** PDX with moderate STn expression within the membrane and the cytoplasm of cancer cells, both in P0 and P4. **(C)** PDX with high cytoplasmic and membranous STn expression, both in P0 and P3. **(D)** PDX with a decrease in STn expression with successive engraftment cycles. **(E, F)** PDXs with moderate STn expression in the initial engraftment (P0), with an increase in subsequent passages.

Sialyl-Tn expression was assessed in PDAC PDXs to investigate whether this short *O*-glycan is expressed after grafting patient tumor cells into a new microenvironment. Assessments were performed immediately after engraftment and tumor growth (passage 0), and after successive engraftments and tumor growth cycles (passage 1-4). Among the 12 PDXs tested, only 2 (16.7%), specifically PDX 8 and PDX 12, showed no STn expression (**Supplementary Table 1**). Importantly, the majority of PDXs maintained STn expression across passages, both in terms of the percentage of tumor area and the localization of the staining (**Figure 3**; **Supplementary Table 1**). As evidenced in **Figure 3**, STn-positive PDXs exhibited significant intra- and inter-tumoral heterogeneity in STn expression levels. Some PDXs showed moderate STn staining (**Figures 3A, B**), while others displayed very strong and uniform positive staining for STn (**Figure 3C**). The staining patterns also varied, with some cases showing exclusive cytoplasmic staining (**Figure 3A**), and others exhibiting both membranous and cytoplasmic staining (**Figures 3B, C**). One case demonstrated a significant decrease in STn expression with successive engraftment cycles, with an initial membranous localization shifting towards a more cytoplasmatic pattern (**Figure 3D**; **Supplementary Table 1**). Additionally, two large duct mucinous tumors that exhibited moderate STn expression in the first engraftment displayed increased STn levels in subsequent passages (**Figures 3E, F**; **Supplementary Table 1**).

## 4 Discussion

Pancreatic cancer remains a significant clinical burden worldwide, characterized by a poor survival rate (1, 2). This dismal outlook can be attributed to several factors, including the silent clinical progression of PC, the lack of early diagnostic tools, and the limited efficacy of currently available treatments (3, 4). Recent advances in immunotherapeutic approaches, such as ADCs and CAR T cell therapy, have shown promise in the cancer field (55, 56). Tumor-associated glycan antigens have emerged as potential molecular targets for immunotherapy due to their distinctive cell surface presentation and restricted expression patterns in malignant cells (57). In particular, the truncated *O*-glycan STn antigen stands out due to its high expression in epithelial-origin tumors, including PC, while being mostly absent in normal tissues (10–13, 15–21). This cancer-associated expression profile has positioned STn as a compelling therapeutic target for various therapeutic strategies, including radiolabeled antibodies, ADCs, and CAR T cells (**Supplementary Table 2**). Nonetheless, the challenge in accurately mapping and targeting STn arises from the variability in binding properties of available mAbs, which has impeded the approval of STn-targeted therapies. Current research on STn in PC has primarily used the HB-STn and TKH2 mAbs for detection (10, 17, 45–48, 58). However, there has been limited investigation into one of the most established anti-STn mAbs, B72.3, despite its extensive use in tissue-based, serologic, and preclinical assays (49, 50) (53, 59, 60).

In the present study, we performed a comprehensive mapping of STn expression in normal pancreatic tissues, as well as in premalignant and malignant pancreatic lesions, using the B72.3 mAb. Our results demonstrated a notable absence of STn in normal pancreatic tissues, consistent with previous studies (10, 17, 45, 46, 48, 59, 60). In premalignant pancreatic lesions, STn expression was observed in PanIN lesions but not in chronic pancreatitis. Previous studies have reported diverse findings regarding STn expression within premalignant lesions of the pancreas, ranging from absence to moderate expression (10, 47, 53, 59). The present study reinforces the need for further validation using a larger independent cohort to clarify the role of STn in pancreatic lesions and assess its potential as a biomarker for the early detection of PC.

In the context of PDAC, the most prevalent type of PC, accounting for more than 90% of all pancreatic malignancies, our study revealed the presence of STn in 61.5% of the cases. Our results align with previous studies reporting that STn expression in PDAC is highly variable (10, 17, 45–48, 53, 59, 60). This variability highlights the challenges involved in precisely detecting short *O*-glycans, potentially arising from different antibody specificities, each recognizing particular epitopes of the STn antigen, influencing detection outcomes across studies. Moreover, disparities in sample size, demographic characteristics of patients, and stages of disease progression within the studied populations may further contribute to the observed differences in STn levels. Heterogeneity in STn expression was observed not only among different tumors but also within individual tumor lesions. Intra-tumor heterogeneity suggests that subsets of tumor cells may express different STn levels. This is consistent with previous studies documenting the heterogenous nature of STn expression in PDAC (48, 59). Considering the poor clinical prognosis linked to STn-positive tumors and the oncogenic features induced by *O*-glycan truncation (11, 12, 22, 61, 62), these observations suggest that short *O*-glycans may play a role in a subpopulation of tumor cells, potentially contributing to cancer progression. Regarding the staining pattern of STn within PDAC, we observed the presence of this glycan both in the cytoplasm and on the membrane of cancer cells. The expression of STn on the surface of cancer cells is crucial for effective targeting, emphasizing its potential as a promising therapeutic target in PC.

Additionally, our study revealed the presence of STn in rare PC subtypes, such as pancreatic acinar cell carcinoma and adenosquamous carcinoma. Although STn detection in these rare pathological subtypes of PC suggests its potential as a biomarker and indicates possible avenues for exploring immunotherapeutic approaches, further validation in larger, more comprehensive cohorts is warranted to validate STn expression across rare PC subtypes and to assess its broader clinical relevance.

We further evaluated the potential association between STn expression and clinicopathological variables, revealing a correlation between STn status and pathological diagnosis, as expected. Notably, higher STn expression was observed in patients with stage II PDAC compared to those with stage I tumors. To our knowledge, this is the first evidence of an association between STn expression levels and PDAC staging. These findings suggest that STn may serve as a marker of tumor progression and could potentially hold prognostic implications in PDAC. However, our study did not reveal a correlation between STn status and OS in an exploratory cohort. Given the relatively small size of this exploratory cohort, future research involving larger cohorts with comprehensive follow-up data is warranted to assess STn’s potential as a prognostic marker for survival in PDAC patients.

To validate our findings and explore the potential of STn for therapeutic applications, STn expression was assessed within a preclinical PDX model of PDAC. Among the 12 PDXs evaluated, 10 exhibited positive STn expression (83.3%). Notably, most PDXs consistently sustained STn expression across passages, both in terms of the extend of stained tumor area and the subcellular staining location. This suggests that STn-expressing cancer cells persist following engraftment. However, some variations were observed: one case showed a decrease in STn with successive engraftment cycles, while two PDXs exhibited increasing STn expression in subsequent passages. The dynamic interplay with the tumor microenvironment may contribute to the different STn levels found in tumor cells, highlighting the importance of this truncated *O*-glycan structure in the cancer context (63, 64). Similar to PDAC patient tissues, variations in staining intensity and patterns were noted between different PDXs, confirming their representativeness of STn expression within the PDAC landscape. The consistent expression of STn in PDX models highlights their potential as valuable tools for preclinical studies and testing of STn-targeted therapies. To our knowledge, this study presents the first evidence of STn mapping in PDAC PDXs, highlighting their utility as preclinical models.

Overall, our study provides a comprehensive evaluation of STn expression using the B72.3 mAb across pancreatic tissues, from normal to premalignant and malignant lesions, and extending to a preclinical model. Despite the inherent challenges associated with mapping STn due to antibody specificity and variability, our findings offer valuable insights into its expression dynamics throughout different stages of pancreatic carcinogenesis. Moreover, our evaluation of STn in rare PC subtypes expands its potential as both a biomarker and therapeutic target for these specific PC subtypes. Additionally, our analysis of STn expression in PDAC PDX models underscores their reliability for preclinical studies and the development of STn-targeted therapies.

## Data Availability

The raw data supporting the conclusions of this article will be made available by the authors, without undue reservation.
