# Supplementary material for: Sialyl-Tn glycan epitope as a target for pancreatic cancer therapies
Source: Front Oncol. 2024 Sep 13;14:1466255. doi: 10.3389/fonc.2024.1466255 (PMC11427427; doi:10.3389/fonc.2024.1466255)
Supplement: Supplementary file 1 [file Table1.docx]

**Supplementary Table 1. Sialyl-Tn expression in a preclinical patient-derived xenograft model of pancreatic ductal adenocarcinoma.**

| **PDX** | **Passage** | **STn expression (%)** |
| --- | --- | --- |
| 1 | 2 | 50-75 |
| 2 | 0 | 5-25 |
| 3 | 0 | 50-75 |
|  | 4 | 50-75 |
| 4 | 0 | 5-25 |
|  | 2 | 5-25 |
| 5 | 0 | 50-75 |
|  | 3 | 50-75 |
| 6 | 0 | 75-100 |
|  | 4 | 5-25 |
| 7 | 0 | 5-25 |
|  | 3 | 50-75 |
| 8 | 0 | <5 |
|  | 3 | <5 |
| 9 | 0 | 5-25 |
|  | 3 | 5-25 |
| 10 | 0 | 5-25 |
|  | 1 | 5-25 |
| 11 | 0 | 5-25 |
|  | 1 | 50-75 |
| 12 | 0 | <5 |
|  | 1 | <5 |

Abbreviations: PDAC, pancreatic ductal adenocarcinoma; PDX, patient-derived xenograft; STn, Sialyl-Tn.
