# Supplementary material for: Sialyl-Tn glycan epitope as a target for pancreatic cancer therapies
Source: Front Oncol. 2024 Sep 13;14:1466255. doi: 10.3389/fonc.2024.1466255 (PMC11427427; doi:10.3389/fonc.2024.1466255)
Supplement: Supplementary file 2 [file Table2.docx]

**Supplementary Table 2. Overview of Sialyl-Tn-based therapeutic approaches.**

| **Type of therapy** | **Construct** | **Condition** | **Reference** |
| --- | --- | --- | --- |
| Vaccine | STn-KLH | Breast cancer | [29, 30] |
|  | STn(c)-KLH | Breast cancer | [31] |
| Radiolabeled antibody | ^111^In-B72.3 | Colorectal cancer | [32, 33] |
|  | ^90^Y-B72.3 | Ovarian cancer | [34] |
|  | ^131^I-CC49 | Prostate and colorectal cancer | [24, 35] |
|  | ^177^Lu-CC49 | Ovarian cancer | [36, 37] |
| ADC | CC49-MMAE | Ovarian cancer | [25] |
|  | 2G12-2B2-MMAE | Ovarian cancer | [26, 38, 39] |
| CAR T cell | B72.3-based scFv | Gastrointestinal cancer | [49] |
|  | CC49-based scFv | Colorectal and ovarian cancer | [27,40-42] NCT05225363 |
|  | L2A5-based scFv | Breast and bladder cancer | [44] |

Abbreviations: ADC, antibody-drug conjugate; CAR, chimeric antigen receptor; ^131^I, Iodine-131; MMAE, monomethyl auristatin E; ^177^Lu, Lutetium-177; STn, Sialyl-Tn; ^90^Y, Yttrium-90.
